# Caliper measurement to improve assessment of neck lumps: comment 2

**DOI:** 10.1308/003588413X13511609956570

**Published:** 2013-01

**Authors:** G Alex

**Affiliations:** United Lincolnshire Hospitals NHS Trust,UK

I read with interest the article by Wesson *et al* and would like to respond to the authors’ conclusion that ‘Caliper measurement is more accurate than clinical palpation’. I fail to understand why research is needed with concurrent waste of time, effort and resources to establish something that is so obvious.

